# Determining Front-Line Therapeutic Strategy for Metastatic Clear Cell Renal Cell Carcinoma

**DOI:** 10.3390/cancers14194607

**Published:** 2022-09-22

**Authors:** Kevin K. Zarrabi, Oladimeji Lanade, Daniel M. Geynisman

**Affiliations:** 1 Sidney Kimmel Cancer Center, Department of Medical Oncology, Thomas Jefferson University, Philadelphia, PA 19107, USA; 2 Fox Chase Cancer Center, Department of Medical Oncology, Temple University Health System, Philadelphia, PA 19111, USA

**Keywords:** renal cell carcinoma, targeted therapy, immunotherapy

## Abstract

**Simple Summary:**

Kidney cancer occurs more commonly within the general population and has historically been associated with morbidity and early death. The most common form of kidney cancer is clear cell renal cell carcinoma. Thankfully, there now have multiple new therapies available for patients with metastatic disease that are improving the lives of patients. However, the number of new drugs and treatment types has created a degree of complexity in treatment planning. We review the most recent data in the metastatic disease space surrounding treatment for patients with newly diagnosed metastatic clear cell renal cell carcinoma. These treatments include surgery, radiation, and systemic therapies. Current systemic therapies include drugs that activate the immune system to target cancer cells and small-molecule drugs that target a cancer cell’s ability to grow and survive.

**Abstract:**

The therapeutic landscape for metastatic renal cell carcinoma has rapidly evolved over the years, and we are now in an era of combination therapy strategies employing immune checkpoint blockade and anti-angiogenesis targeted therapy. Since 2018, we have gained regulatory approval for four distinct combination therapies, all with survival benefits, and with guideline recommendation for use in the front-line setting. As such, treatment selection has become increasingly complex with a myriad of treatment choices but little high-level head-to-head data to guide treatment selection. Heterogeneity in tumor biology further complicates treatment selection as tumors vary in behavior and treatment responsiveness. Ongoing development of biomarkers will certainly assist in this setting, and validation of predictive markers represents an unmet need. In their absence, we highlight features of disease and nuances to datasets from landmark prospective clinical trials to help inform treatment selection. There is growing evidence to support deferring upfront systemic therapy in some patients, with opportunities for active surveillance or metastasis-directed therapy. In others, upfront systemic therapy is warranted and necessitates thoughtful consideration of multiple clinicopathologic parameters to inform optimal patient-centered decision making.

## 1. Introduction

Renal cell carcinoma (RCC) is the eighth most common malignancy in the United States, with an annual incidence on the rise [1]. RCC is a biologically diverse tumor with varying drivers of tumorigenesis, and with >75% of tumors being clear cell in histology. Advanced disease had been traditionally associated with morbidity and early mortality as tumors proved resistant to conventional radiotherapy, hormonal therapy, and cytotoxic chemotherapy. Prior to 2005, the mainstays of therapy were high-dose interleukin-2 (HDIL-2) and interferon-alpha (IFN-α). In this setting, treatment toxicities were abundant and outcomes were generally poor with durable responses observed in the minority of patients. However, over the last 20 years we have observed a paradigm shift in the treatment of metastatic RCC (mRCC) with the emergence of novel therapeutic approaches that have dramatically improved survival [2].

The discovery of the von Hippel–Lindau (VHL) tumor suppressor gene, and its ubiquitous loss of function in clear cell disease has improved our understanding of the underlying molecular drivers of RCC. VHL encodes for the substrate of the ligase that targets hypoxia-inducible factor (HIF) for destruction in the presence of oxygen [3]. When function is aberrant, there is unregulated expression of vascular endothelial growth factor (VEGF) and its downstream signaling cascade. This leads to unregulated cell growth, angiogenesis, and tumor cell invasion [4]. Targeting this pathway has served as a therapeutic strategy and has allowed for a ‘revolution’ in drug development for mRCC with multiple targeted therapies available that have improved survival.

Capitalizing on the immunogenicity of RCC, and based on prior success with HDIL-2 and IFN-α, we witnessed a second ‘revolution’ in the mRCC treatment paradigm with the advent of immune checkpoint blockade (ICB). ICB includes agents targeting cytotoxic T-lymphocyte-associated protein 4 (CTLA-4), programmed cell death protein 1 (PD-1), and programmed death-1 ligand 1 (PD-L1). Immuno-oncology (IO)-based strategies are now employed in the front-line setting for mRCC. Patient outcomes continue to improve and there has been a dramatic shift in median survival from being <1 year in the 1990s to over 4 years in most recent trials [5].

These successes have led to approvals in consecutive years of a number of agents as monotherapy or in combination strategies. The rapid rate of discovery and drug development for mRCC has led to approval of a myriad of treatment options that has created a degree of complexity that has yet to be reconciled in the literature. We now have United States Food and Drug Administration (FDA) approval and guideline recommendation for four combination therapies in the front-line setting (Table 1). Cross-trial comparisons are a challenge here, as the major studies employ varying end points, statistical strategies, and populations. We provide an overview of the data supporting a variety of treatment approaches, and highlight nuances that may assist in clinical decision making.

## 2. Risk Stratification to Guide Management

Prior to considering systemic therapy, applying clinically validated tools to risk stratify the patient’s disease is recommended. The International Metastatic RCC Database Consortium (IMDC) criteria are a validated prognostic model, which categorizes patients by risk of early cancer mortality (three risk groups: favorable, intermediate, or poor) [12]. The IMDC criteria consists of 6 clinical parameters; anemia, thrombocytosis, Karnofksy performance status, hypercalcemia, neutrophilia, and time from diagnosis to initiation of systemic therapy. Nearly 80% of patients encountered in clinical practice have IMDC intermediate- or poor-risk disease [13]. Many of the landmark trials that inform guideline recommendations integrated the IMDC risk model into cohort assignment as well as statistical analyses. As such, accounting for IMDC risk is necessary for applying standard-of-care therapy.

The IMDC criteria was designed during the development of anti-VEGF targeted therapies. As such, its value nests in the angiogenesis-driven biology of RCC tumors. Now in the contemporary era of IO, the criteria continue to demonstrate prognostic and predictive value and it is believed that the criteria reflect the multifaceted underlying biology within the RCC tumor microenvironment and that they remain applicable [14,15]. Ongoing studies are integrating markers of immunogenicity and genomics into risk stratification [16,17], but these have not yet been incorporated into standard practice recommendations.

## 3. Deferring Initiation of Systemic Therapy

### 3.1. Active Surveillance

There is a growing body of evidence supporting the use of active surveillance (AS) in select patients with metastatic disease. In patients with indolent growth and who are asymptomatic from tumor burden, AS provides an opportunity for a prolonged toxicity-free interval without compromising survival. This was formally investigated in a prospective phase II trial, which enrolled 52 treatment-naïve, asymptomatic patients with mRCC. Patients were followed radiographically until initiation of systemic therapy, the timing of which was at the discretion of the treating physician and patient [6]. At a median follow-up time of 38.1 months, the median time of surveillance was 14.9 months. The trial investigators performed a subset analysis whereby the study population was partitioned into a favorable group (0–1 IMDC risk factor and ≤2 metastases) and an unfavorable group (IMDC 2–6 risk factors, >3 metastases). The favorable group had a remarkable median surveillance time of 22.2 months (95% CI, 13.8–33.3) compared to 8.4 months (3.2–14.1; *p* = 0.0056) for the unfavorable group. Remaining on AS had no adverse effects on quality of life, including anxiety and depression. A subsequent prospective observational study of 143 patients further supported the role for AS in mRCC. In the MaRCC trial, 143 patients were started on AS and were followed for a median of 33.0 months. A total of 70 patients remained on AS at data cut off and the median overall survival (OS) was not reached [7]. Altogether, there is ample data to support the role of AS in a subset of patients with indolent tumor biology and favorable risk disease. Thus, making sure a patient actually needs any treatment at all for their advanced RCC is the first question to always address.

### 3.2. Cytoreductive Nephrectomy Followed by Active Surveillance

The role for cytoreductive nephrectomy (CN) remains heavily debated, and there have been recent changes to standard practices with decreasing support for upfront nephrectomy. The data that supported CN stemmed from the early IO era when studies demonstrated both a progression-free survival (PFS) and OS benefit in those who underwent CN prior to initiating systemic therapy with IFN-α [8,18]. This practice then came into question as we developed a more nuanced understanding of the heterogeneity in RCC tumor biology and in the era of more modern therapies (TKI/ICB). The more contemporary CARMENA and SURTIME trials were performed in the era of targeted therapies and also account for the IMDC risk criteria. The CARMENA trial was a non-inferiority randomized control study investigating CN followed by sunitinib or upfront sunitinib in 450 IMDC intermediate- or poor-risk patients. The median OS was 18.4 months with sunitinib alone and 13.9 months for CN followed by sunitinib, indicating non-inferiority (hazard ratio (HR) 0.89; 95% CI, 0.71–1.10) [9]. The SURTIME trial randomized patients to CN followed by sunitinib or deferred CN after three cycles of sunitinib. The study did not accrue as planned, and was closed prematurely. However, at 28-week PFS analysis, there was no appreciable difference in survival between the two groups. Interestingly, there was an OS advantage in the deferred CN group in the intention-to-treat (ITT) analysis (32.4 m vs. 15.0 m; HR 0.57; 95% CI, 0.34–0.95; *p* = 0.03) [19]. Together, these trials suggest that upfront CN should be considered only in carefully selected patients, and that starting with systemic therapy may confer overall benefit for the mRCC population at large.

We can extrapolate that upfront CN may benefit select patients with few IMDC risk factors, asymptomatic disease, and minimal metastatic tumor burden [20]. This, however, is the same patient profile that can benefit from AS, and there is no prospective data to guide decision making between surgery or AS. In both large AS studies, a subset of patients had undergone CN prior to AS (or underwent CN during the AS interval). Unfortunately, the studies do not provide survival analyses in relation to these patient subsets. As such, we recommend a multidisciplinary discussion taking patient wishes and goals into account as both AS and upfront CN are viable treatment strategies.

There is growing interest in identifying the ideal patient population for deferred CN and ongoing studies aim to further refine our understanding of CN. A number of large-dataset analyses demonstrated a longer median OS in carefully selected patients with TKI treatment and deferred CN [21,22]. A recent real-world analysis evaluated the oncologic role of CN in the modern era of IO, and identified benefit with CN in select patients as well [23]. Thankfully, we have two prospective phase III randomized control trials underway examining deferred CN in the modern era of IO. Neither study design includes an upfront CN cohort, which speaks to the diminishing role of this practice. The NORDICSUN (NCT03977571) study is examining outcomes in select patients (≤3 IMDC risk features) treated with ipilimumab/nivolumab and deferred CN. The PROBE (NCT04510597) study allows for multiple front-line systemic therapy options, and if no disease progression, then patients may proceed with a deferred CN. Both studies are highly anticipated and will likely shed light on potential survival benefit to systemic therapy alone.

### 3.3. Upfront Metastasis-Directed Therapy

Metastasis-directed therapy (MDT) should always be a consideration in the event of brain metastases, extreme pain and cord-compression (or impending cord compromise). Although TKI therapy can induce rapid disease control and is an attractive option, one must consider the risk of delayed perioperative intervention due to wound healing or radionecrosis and compound toxicity. In absence of these extreme circumstances, MDT with radiotherapy or surgery can be considered depending on degree of tumor burden and patient comorbidities.

Although RCC has been traditionally considered resistant to radiotherapy, emerging data is changing this opinion. Conventionally fractioned radiotherapy was previously examined in RCC with seemingly little antitumor activity [24]. (SBRT) provides a higher dose of radiation while sparing adjacent healthy tissue from toxicity. This higher dose of radiation is believed to overcome the theoretical radioresistance mechanisms of RCC [25]. A meta-analysis of 28 studies accounting for 1602 mutually exclusive patients evaluated the safety and efficacy of SBRT for RCC oligometastases. Although with significant limitations and not directly applicable to the treatment-naïve population, the study concluded that stereotactic radiotherapy was safe and efficacious with a local control rate of 90% and negligible toxicity at 12 months [26]. In a prospective phase II study, definitive SBRT was assessed as a means to defer systemic therapy in patients with oligometastatic mRCC. The study enrolled 30 patients who underwent at least one round of radiotherapy. At a median follow-up of 17.5 months, the PFS was 22.7 months (95% CI, 10.4–NR), and the 12-month PFS was 64% (95% CI, 48–85) [10]. The role of SBRT as an initial therapeutic modality is growing, and further studies are likely to further define the role of SBRT in the mRCC treatment paradigm.

Surgical resection or oligometastasectomy provides an alternative approach to newly diagnosed mRCC in select patients. There is no high-level prospective data comparing oligometastasectomy to AS or SBRT. However, opportunity for resection may be encountered in clinical practice, and a multidisciplinary review of these cases is warranted. In an observational study of complete metastasectomy at first recurrence after partial or radical nephrectomy, the two-year cancer-specific survival was significantly higher in patients with surgery to those without (84% vs. 54%; *p* < 0.001) [11]. Similar results were found in studies that included resection of metastases to the lung, bone, brain, liver, and pancreas [27,28]. Possibly the most compelling data supporting metastasectomy comes indirectly from the literature exploring adjuvant therapy. The KEYNOTE-564 trial investigated adjuvant pembrolizumab in patients with locoregional RCC with high risk of recurrence. The study included a subset of patients who had resection of metastatic lesions conferring a no evidence of disease state (M1 NED) [29]. Treatment with adjuvant pembrolizumab in this population had a profound impact on DFS (HR 0.28, 95% CI 0.12–0.66). Although the role of adjuvant IO remains unclear with inconsistent results among large prospective trials [30,31], metastasectomy conferring M1 NED may serve an opportunity for prolonged survival in select patients planned for adjuvant therapy.

## 4. Front-Line Systemic Therapy Options

### 4.1. IO/IO Combination Therapy

The CheckMate 214 study led to approval of ipilimumab/nivolumab combination therapy for IMDC intermediate- or poor-risk patients. CheckMate 214 was a phase III randomized control trial investigating ipilimumab/nivolumab or sunitinib in treatment-naïve patients. Coprimary end points were ORR, PFS, and OS among intermediate- and poor-risk patients. Exploratory end points included outcomes according to the tumor PD-L1 expression (≥1% vs. <1%). The study enrolled 1096 patients in the ITT population. The pre-defined statistical analyses were focused on IMDC intermediate- and poor-risk patients, although the study included 249 favorable risk patients as well. Median follow-up time was 25.2 months at time of initial analysis, and ORR was 42% (95% CI, 37.0–47.0) with ipilimumab/nivolumab versus 27% (95% CI, 22.0–31.0) with sunitinib (*p* < 0.001). There was no median PFS benefit to the IO/IO combination therapy as the hazard ratio for disease progression or death did not meet prespecified threshold for statistical significance (HR 0.82; 99.1% CI, 0.64–1.05; *p* = 0.03). The median OS was not reached (NR) with ipilimumab/nivolumab versus 26.0 months with sunitinib (HR for death, 0.63; *p* < 0.001). In the favorable risk population, the OS, ORR, and PFS all favored sunitinib over ICB combination therapy. Grade 3/4 treatment-related adverse events (TRAE) occurred in 46% of ipilimumab/nivolumab patients and 63% of sunitinib-treated patients. High-dose glucocorticoid for immune-related adverse events (irAE) were needed in 35% of ipilimumab/nivolumab patients with a TRAE [32]. Based on this landmark study, the FDA approved ipilimumab/nivolumab for first-line treatment of IMDC intermediate- and poor-risk patients in April 2018.

The CheckMate-214 was the first combination therapy in the modern era to report an OS benefit. We now have extended follow up survival data from the study reported (Table 2). At 5-year follow-up, dual-ICB continues to demonstrate an improved median OS over sunitinib in the ITT (HR 0.72; 95% CI, 0.62–0.85) and IMDC intermediate-and poor-risk (HR 0.68; 95% CI, 0.58–0.81) populations. PFS probabilities also favored ipilimumab over sunitinib in intermediate-and poor-risk patients (HR 0.73; 95%, CI 0.61–0.87). However, in favorable risk patients, OS and PFS both favored sunitinib (OS: HR 0.94; 95% CI 0.65–1.37; PFS: HR 1.60; 95% CI 1.1–2.3) [33].

### 4.2. IO/TKI Combination Therapy

The early individual success of nivolumab monotherapy and of older-generation targeted therapies naturally led to the investigation of IO/TKI combination strategies. In fact, early data suggest that anti-angiogenesis therapy promotes antitumor immune responses within the tumor microenvironment, and combination approaches mutually enhance the effects of anti-angiogenesis and IO therapy [34]. The first landmark IO/TKI combination trial was the KEYNOTE-426 study, which employed axitinib in combination with pembrolizumab or sunitinib in 861 treatment-naïve patients. This combination therapy was approved by the FDA in April 2019, and has the longest follow-up data among all IO/TKI regimens. The primary end points were OS and PFS in the ITT population. The key secondary end point was the ORR. At time of initial reporting after a median follow-up of 12.8 months, survival analysis favored axitinib/pembrolizumab over sunitinib (HR 0.53; 95% CI, 0.38–0.74; *p* < 0.0001). Median PFS for axitinib/pembrolizumab and sunitinib-treated patients were 15.1 months and 11.1 months, respectively (HR 0.69; 95% CI, 0.57–0.84; *p* < 0.001). ORR was 59.3% (95% CI, 54.5–63.9) in the axitinib/pembrolizumab group and 35.7% (95% CI, 31.1–40.4) in the sunitinib group (*p* < 0.001). Importantly, the superior outcomes from with IO/TKI combination therapy were observed across all IMDC risk groups [35]. With extended follow up, and a median follow-up of 30.6 months, a continued clinical benefit was observed with combination therapy in OS (HR 0.68; 95% CI 0.55–0.85, *p* = 0.0003) and PFS (15.4 months vs. 11.1 months, *p* < 0.0001) [36].

Following a similar trial design, the CheckMate 9ER study randomized 651 treatment-naïve patients with clear cell mRCC to cabozantinib/nivolumab or sunitinib. The primary endpoint was PFS. Secondary endpoints were OS and ORR. The median PFS was 16.6 months (95% CI, 12.5–24.9) for cabozantinib/nivolumab and 8.3 months (95% CI, 7.0–9.7) for sunitinib. The OS (HR 0.60; 98.89% CI, 0.40–0.89; *p* = 0.001) and ORR (55.7% vs. 27.1%; *p* < 0.001) also favored the combination regimen. These benefits were noted across IMDC risk groups [37] (Table 2).

Most recently, a third IO/TKI combination regimen gained FDA approval for use in mRCC. The CLEAR study demonstrated the efficacy of lenvatinib/pembrolizumab combination therapy. The phase III randomized trial assigned 1069 patients in a 1:1:1 ratio to lenvatinib/pembrolizumab, lenvatinib/everolimus or sunitinib. The study’s primary endpoint was PFS, and secondary endpoint was OS. Lenvatinib/pembrolizumab demonstrated a significantly longer PFS than sunitinib in the ITT population (median PFS 23.9 vs. 9.2 months; HR, 0.39; 95% CI, 0.32–0.49; *p* < 0.001). Median OS was not reached in either treatment arm, but the OS was longer with lenvatinib/pembrolizumab than with sunitinib (HR 0.66; 95% CI, 0.49–0.88; *p* = 0.005) (Table 2). Importantly, the CLEAR data reported an ORR of 71% and a CR rate of 16% in lenvatinib/pembrolizumab treatment patients [38].

Three alternative combination therapies have been studied and may be considered for use, although they are not guideline-recommended as front-line choices. The JAVELIN Renal 101 trial was a phase III study comparing avelumab/axitinib with sunitinib. The study focused on PD-L1-positive tumors, with coprimary endpoints of PFS and OS in this biomarker subset [39]. Extended follow-up results demonstrated benefit to avelumab/axitinib over sunitinib across IMDC risk strata for ORR, but only benefit in for PFS and OS among IMDC intermediate/poor-and poor-risk disease [40]. An alternative IO/TKI combination therapy with emerging efficacy data is axitinib/nivolumab. A phase I/II study investigating this combination in treatment-naïve patients and patients previously treated with TKIs alone or IO/IO combination. In the treatment-naïve group, ORR was 69.0% and a disease control rate (DCR) of 97.6%. Median PFS was 16.4 months (95% CI, 10.6–21.9) and median OS was not reached [41].

## 5. Factors to Consider with Front-Line Therapy Options

### 5.1. Opportunity for Multidisciplinary Approach

Prior to deciding between systemic therapy options, we recommend evaluating patient candidacy for active surveillance or metastasis-directed therapy. Metastatic RCC remains largely incurable, and studies have demonstrated the safety and added value (in QoL and survival) of AS in select patients. We strongly recommend considering AS for favorable risk patients with limited tumor burden and indolent growth. The CARMENA trial showed possible benefit for CN in a patient subset with similar characteristics. To this end, some patients undergoing AS can also be considered for CN (either upfront or during the AS period), but we generally caution against this practice at least until more data emerges from ongoing trials. However, we do consider CN in symptomatic patients who may have uncontrolled hematuria or localized pain from the primary tumor. In the setting of oligometastatic disease with overall indolent tumor biology aside from 1 to 2 growing lesions, metastasis-directed therapy with metastasectomy or SBRT are reasonable approaches with emerging supporting data. Taken together, an individualized approach to each patient is recommended, and if there is opportunity to avoid or defer systemic therapy, a multidisciplinary review of the case is appropriate.

### 5.2. Characterization of the Tumor Biology

Clinical decision making should account for tumor biology. Sarcomatoid differentiation appears in approximately 8% of clear cell tumors but is present in nearly 20% of metastatic RCC [42,43]. The sarcomatoid disease is typically more aggressive and historically responds poorly to anti-angiogenesis therapy. In the CheckMate 214 trial, 139 patients with intermediate- and poor-risk disease had sarcomatoid histology. In this subset of patients, median OS was not reached with ipilimumab/nivolumab and was 14.2 months with sunitinib (HR 0.45; 95% CI, 0.3–0.7; *p* = 0.0004). Median PFS also heavily favored ipilimumab/nivolumab (median 26.5 vs. 5.1 months; HR, 0.54 (95% CI, 0.33–0.86; *p* = 0.0093)). The most pronounced benefit was observed in ORR, which was 60.8% (complete response 18.9%) for ipilimumab/nivolumab and 23.1% (complete response 3.1%) with sunitinib. [44]. Subgroup analyses from the KEYNOTE-426, CheckMate9ER, and CLEAR studies have also shown substantial benefits from IO/TKI combination therapy over TKI monotherapy (Table 3). Meta-analysis of patients with sarcomatoid histology from multiple ICI-based randomized control trials also demonstrates this benefit in OS, PFS, ORR, and complete response (CR) rate [45]. In the absence of a contraindication to IO, we believe all patients with clear cell mRCC with sarcomatoid differentiation should be treated with a front-line ICB-based systemic therapy regimen. Ipilimumab/nivolumab remains the regimen with the longest follow-up and with the highest ORR and CR rate within this population.

PD-L1 expression as a predictive biomarker has been extensively studied in mRCC. In the CheckMate 214 study, ipilimumab/nivolumab showed a PFS benefit over sunitinib in PD-L1-positive tumors, but not in PD-L1-negative tumors (PD-L1 < 1%). However, in the IMDC intermediate-and poor-risk patients, PD-L1 expression did not correlate with response. IO/TKI therapy demonstrated benefit regardless of PD-L1in the KEYNOTE-426, CheckMate 9ER, and CLEAR studies. The studies were not consistent in regards to definition for PD-L1 positivity and employed different commercial assays for PD-L1 in each respective study. At this time, PD-L1 as not a clinically validated predictive biomarker in mRCC and we do not incorporate its expression in our routine decision making.

Considering whether there are genomic contributions at play is important since identifying a hereditary RCC syndrome can occasionally affect clinical management and clinical trial participation. The National Comprehensive Cancer Network guidelines recommend genetic risk evaluation in all RCC patients who meet prespecified criteria, namely that they (1) have a close blood relative with a known pathogenic variant in a cancer susceptibility gene, (2) have been diagnosed at age <46 years, (3) have bilateral or multifocal tumors, (4) have ≥1 first- or second-degree relative with RCC, and (4) have tumors that are characterized by certain specified histologic characteristics [46]. Identification of a genetic syndrome or germline gene may also warrant expert pathology review of tumor tissue, as rare histologies can be mistakenly identified as clear cell histology when they actually represent a rarely encountered histologic subtype necessitating alternative treatment strategy.

### 5.3. Patient Characteristics and Toxicity

Anticipating a patient’s ability to tolerate therapy is essential to treatment planning. Avoiding dose reductions and treatment interruptions should be a determinant in choice of therapy. Front-line combination therapy remains standard of care at this time, and we believe that all patients should be considered for an ICB-based doublet therapy (IO/IO or IO/TKI) in the absence of contraindication or significant co-morbidity. History of autoimmune disease or solid-organ transplant remain the strongest considerations in avoiding IO. In these cases, TKI monotherapy can still provide effective disease control with durable outcomes in some. There is robust data supporting the use of cabozantinib monotherapy, especially in IMDC intermediate-and poor-risk patients where it has superiority over other single-agent anti-angiogenesis therapy [37]. Sunitinib remains a reasonable TKI to consider in some IMDC favorable risk patients who are not candidates for IO, especially considering there is no proven OS benefit here over dual-ICB therapy to date [37].

TKI therapy is associated with significant toxicity. The landmark IO/TKI studies had higher incidence of grade ≥ 3 toxicities than in CheckMate 214, most of which are attributable to the TKI. These toxicities are predictable, and typically resolve with dose reduction or treatment breaks. However, poor wound healing, vascular disease, severe coronary artery disease, recent CVA, or uncontrolled hypertension all represent co-morbid conditions, which may be barriers to safe TKI use altogether. Dual or sometimes single-ICB therapy represents an ideal treatment consideration in this patient subset.

The nature of the toxicities differ between IO and TKI classes, and these toxicities should be taken into consideration during patient-centered decision making. The majority of toxicity incurred from IO/TKI combination therapy is attributable to the TKI. Although TKI-associated toxicity is common, it is predictable and can be mitigated with preventive measures, dose reductions, or treatment breaks. Some patients may choose to avoid the reality of day-to-day TKI-associated toxicity, which affects QoL, and may instead choose dual-ICB therapy. Ipilimumab/nivolumab use has QoL metrics supporting its use over TKI therapy with improved patient-reported outcomes [47]. Conversely, patients with severe end-organ disease (e.g., COPD, CKD) may want to avoid the risk of grade ≥ 3 irAE, which may be life threatening given poor organ reserve. These patients may choose for more day-to-day toxicity from IO/TKI and avoid the 47% risk of grade ≥ 3 irAE from IO/IO, which may cause long lasting morbidity or death.

Financial toxicity remains a factor to consider as certain regimens may burden patients with out-of-pocket costs. Costs associated with mRCC treatments have sharply increased over recent years [48]. Identifying the most cost-effective combination therapy is an area of ongoing study. A recent analysis explored the lifetime cost-effectiveness between ipilimumab/nivolumab, axitinib/pembrolizumab and sunitinib and concluded ipilimumab/nivolumab was the most cost-effective treatment option (accounting for costs of drug treatment, adverse events, and utilities associated with different health states) [49]. Utilizing the data from CheckMate 214 in conjunction with the U.S. 2017 Healthcare Cost and Utilization Project, investigators performed a cost analysis using temporal trends and costs related to grade ≥ 3 TRAE. Treatment with ipilimumab/nivolumab was associated with lower grade ≥ 3 TRAE costs than sunitinib [50]. Ongoing studies will help us better explore the financial toxicity associated with newer therapies, but cost considerations are complex and vary tremendously on a patient-to-patient basis based on insurance coverage or lack thereof. Therefore, we recommend an individualized patient discussion surrounding out-of-pocket expense and overall financial toxicity (including time off work for clinic appointments and infusions, costs associated with potential TRAE, childcare/elder care expenses, and travel costs). These practices are now supported by the ASCO guidelines for management of mRCC [51].

### 5.4. Tumor Burden

In patients with a bulky tumor burden, symptomatic disease, or evidence of end-organ dysfunction, rapid treatment response is necessary. In these settings, ORR and risk of primary progression are clinical endpoints we prioritize. Of all the approved doublet therapies, lenvatinib/pembrolizumab provides the highest ORR (ITT ORR: ipilimumab/nivolumab 39.1%, axitinib/pembrolizumab 60.4%, nivolumab/cabozantinib 54.8%, and lenvatinib/pembrolizumab 71.0%), although the study enrolled the highest proportion of IMDC favorable risk patients. Lenvatinib/pembrolizumab also demonstrated the shortest median time to response (1.94 months), although the CLEAR study protocol-defined timepoint for first response evaluation was at 8 weeks, as opposed to 12 weeks in the other landmark trials of interest. Numerically, lenvatinib/pembrolizumab provides the greatest chance of rapid treatment response, or at least disease control, among front-line options. However, lenvatinib/pembrolizumab-treated patients also have the highest rate of grade ≥ 3 TRAE and drug discontinuation rates among the combination therapies and this treatment regimen has no clear OS advantage over other combination therapies.

Location of metastases with respect to overall tumor burden can also guide therapy. Presence of bone metastases are common in mRCC and are associated with poor prognosis [52]. In pre-clinical prostate xenograft studies, cabozantinib had direct effect on osteoblast activity while inhibiting osteoclasts. In clinical study, cabozantinib led to increased bone scan responses and decreased turnover in prostate cancer patients. This provided the biologic rationale for exploration of skeletal-related events (SREs) in the mRCC setting. Subgroup analysis from the phase III METEOR trial investigating cabozantinib and everolimus in previously treated mRCC suggested cabozantinib may have unique activity against bony disease as survival and response rates were higher and bone turnover was lower in patients with baseline bone metastases [53]. Post hoc analysis from the CheckMate 9ER similarly revealed that combination treatment with cabozantinib/nivolumab had more pronounced efficacy in patients with bone metastases compared to those without [54]. The phase II A031801 (RadiCal) trial through Alliance will employ cabozantinib in combination with Radium-223 or cabozantinib alone in a patients with symptomatic bone metastases. Correlative analyses from this study may better define cabozantinib tropism for bone metastases (NCT04071223). Brain metastases are also challenging to treat and associated with morbidity and early mortality. TKI’s are not conventionally believed to have central nervous system (CNS) activity, however cabozantinib has recent pre-clinical data suggesting blood-brain barrier penetration [55]. A proposed mechanism here is direct targeting of cMET, which is enriched in mRCC brain metastases. In a large and well-designed retrospective analysis of mRCC patients with brain metastases, cabozantinib demonstrated CNS activity with an intracranial response rate of 55% (95% CI, 36–73%) without concurrent brain-directed local therapy [56]. Taken together, cabozantinib/nivolumab represents an ideal candidate for treating patients with baseline bone or brain metastases. Alternatively, ipilimumab/nivolumab’s activity in the CNS was directly assessed in the CheckMate 920 study, which was the first prospective trial of first-line dual-ICB therapy inclusive of patients with brain metastases. The study demonstrated an ORR of 32% (95% CI, 15–54%), although no complete responses. Based on this study, ipilimumab/nivolumab is an alternative regimen to consider for patients with CNS disease.

Axitinib/pembrolizumab represents the IO/TKI option with the most mature efficacy and safety data and is an attractive front-line option. Axitinib/pembrolizumab provides an opportunity for rapid disease control and prolonged survival, with fewer grade ≥ 3 TRAE than other IO/TKI regimens, and fewer treatment discontinuations than lenvatinib/pembrolizumab [35,57]. Further, axitinib is a potent TKI with a short half-life. The short half-life allows for quick washout pre-procedurally when surgical intervention in warranted. Differentiating TKI and IO toxicity is often challenging, but axitinib-related adverse effects typically resolve or improve more rapidly than other TKIs owing to its short half-life. To this end, it is often easier differentiating an irAE from TKI toxicity with axitinib. Axitinib pharmacokinetics (twice daily dosing schedule) along with the 1 mg formulary tablets allow for ease in titration to optimize efficacy, while minimizing adverse effects [58].

Employing axitinib/pembrolizumab in the front-line setting may be advantageous for survival when considering treatment sequencing. Cabozantinib has proven efficacy after prior anti-angiogenesis, and its multitarget activity bypasses mechanisms of resistance to older-generation TKIs such as axitinib and sunitinib. As such, cabozantinib is an ideal second-line agent in patients treated with non-cabozantinib-based front-line combination therapies. Indeed, cabozantinib monotherapy was the most commonly employed second-line therapy after axitinib/pembrolizumab per KEYNOTE-426 [35]. Similarly, lenvatinib-naïve patients may be considered for treatment with lenvatinib/everolimus combination therapy in the second- or third-line settings. Employing axitinib/pembrolizumab upfront leaves these efficacious salvage options available for future use, potentially positively affecting overall survival. However, optimal sequencing is not well defined and requires further investigation.

Ipilimumab/nivolumab use is supported by long-term follow-up data, and has the ongoing appeal of a durable response. We consider ipilimumab/nivolumab in IMDC intermediate-and poor-risk patients who are not in need of a rapid response and can tolerate risk of primary progression. The treatment schedule entails four doses of dual-ICB therapy, and is followed by a very manageable and well-tolerated maintenance nivolumab phase free of daily oral medications and their associated chronic toxicities. In fact, patients treated with ipilimumab/nivolumab had a significantly improved health-related quality of life (HRQoL) over those treated with sunitinib in prospective study [32]. Although durable responses are not exclusive to patients treated with ipilimumab/nivolumab, the long-term follow-up data are exceptional here. The PFS curves in the ITT and IMDC intermediate-and poor-risk populations plateaued at 24-months and have not fluctuated significantly. Moreover, the HR for OS has also remained constant throughout the ongoing extended follow up from CheckMate 214. This is in contrast to follow-up data from all three IO/TKI regimens, which reveal a slowly rising HR suggesting slow but steady loss of treatment effect. Further maturation of the data will certainly clarify long-term survival efficacy, but as of now, ipilimumab/nivolumab provides the most durability in response among approved regimens [5,32,35,36,37,38,57,59].

### 5.5. Employing Real-World Evidence in Lieu of Head-to-Head Data

Cross-trial comparisons are inherently fraught with flawed conclusions, and there is generalized caution against this practice [60,61]. However, in the absence of head-to-head trials among front-line mRCC treatment options, we often rely on data extrapolation. Randomized control trials remain the gold standard for evaluating interventions. However, in attempt to minimize confounders and bias, studies employ strict inclusion and exclusion criteria negatively impacting external validity [62]. Real-world analyses from large datasets can provide evidence that is generalizable and can inform clinical decision making (as well as future trial design and healthcare policy) [63]. A large, retrospective observational study utilizing a longitudinal database with 1506 patients treated with ipilimumab/nivolumab or axitinib/pembrolizumab in both the community and academic settings examined patient outcomes (median follow-up time 20.0 months, range 0.4–47.6 months). The median OS for the full population was 28.9 months for axitinib/pembrolizumab and was 24.3 months for ipilimumab/nivolumab (*p* = 0.09). Twenty-four-month survival was 53.8% for axitinib/pembrolizumab-treated patients and 50.2% for ipilimumab/nivolumab-treated patients. Real-world PFS was 10.6 months for axitinib/pembrolizumab-treated patients and 6.9 months for ipilimumab/nivolumab-treated patients. Interestingly, axitinib/pembrolizumab therapy conferred benefit in PFS and OS in the IMDC favorable risk population [64]. In a separate real-world analysis employing the IMDC database, clinical outcomes were assessed in 723 intermediate-and poor-risk patients treated with ipilimumab/nivolumab or IO/TKI combination therapies. There was no discernable OS difference between treatment groups [65]. The outcomes from these studies are generally concordant with historical trial data, which is noteworthy and reassuring. The blunted efficacy and survival observed in these real-world patients is expected and speaks to the efficacy-effectiveness gap, which typically exists for contemporary cancer therapies [66]. This real-world survival data can certainly assist with bedside patient conversations and prognostication when it comes to choosing therapy.

### 5.6. Can We Consider Ipilimumab/Nivolumab for IMDC Favorable Risk Disease?

Treatment with dual-ICB has distinct benefits to consider in the favorable risk population. Treatment-free survival (TFS) is a clinical endpoint developed in the modern IO era, which characterizes the time free of systemic anticancer therapy, while accounting for ongoing systemic toxicity [67]. In the 42-month follow-up of CheckMate-214, the mean TFS was considerably longer with ipilimumab/nivolumab than sunitinib for intermediate-and poor-risk patients (6.9 vs. 3.1 months). The TFS difference was even more pronounced in favorable risk patients (11.0 vs. 3.7 months). This includes TFS free of toxicity as well [68].

In the IMDC favorable risk cohort from CheckMate 214, considerably more patients achieved a CR with ipilimumab/nivolumab than with sunitinib (12.0% vs. 6.5%) [37]. Moreover, the probability of maintaining a response ≥4 years is higher with ipilimumab/nivolumab in the favorable risk category, 60% (95% CI, 0.41–0.75) vs. 38% (95% CI, 0.22–0.54) with sunitinib. Conditional 5-year survival is now also favoring ipilimumab/nivolumab (63% vs. 55%) [69]. Following current treatment guidelines, however, any patient treated with front-line IO/TKI will likely never receive anti-CTLA-4 therapy in their treatment course. This is unsettling, considering the lost opportunity for a durable response for many of these patients. The phase II HCRN GU16-260 trial investigated the role of salvage ipilimumab as a means of converting suboptimal responses with alternative front-line treatment. Unfortunately, salvage ipilimumab/nivolumab showed limited activity with an ORR of only 11% and only one CR [70]. We may see a growing role for dual-ICB therapy among IMDC favorable risk patients in the future, especially if there is growing sentiment that patients should receive the most active ICB regimen perceived to be tolerable upfront [70].

Another IO option that should not be overlooked for IMDC favorable risk patients is pembrolizumab monotherapy. The KEYNOTE-427 was a multicenter, open-label phase II study of pembrolizumab monotherapy in clear cell mRCC (cohort A). The study enrolled 41 patients with IMDC favorable risk disease. The ORR among these patients was 31.0% (95% CI, 17.6–47.1) and the DCR was 61.9% (95% CI, 45.6–76.4). Notably, the adverse event profile is considerably milder than IO/TKI or IO/IO combination therapy, with a grade ≥ 3 irAE occurring in only 15.1% of patients [71].

Developing IO-specific predictive biomarkers and prediction models can have transformative impact in the treatment paradigm. Interestingly, recent studies categorized mRCC patients by molecular subgroups that predicted treatment responsiveness to IO. There was no correlation between the immunogenic molecular subgroup and IMDC score, and all subgroups were present in all IMDC risk groups [72]. At this time, we do not typically consider IMDC favorable risk patients for dual-ICB therapy or pembrolizumab monotherapy if the patient is a candidate and agreeable to IO/TKI regimens, however further study is certainly warranted to optimize treatment selection in this space.

### 5.7. Prior Treatment with IO in the Adjuvant Setting

The KEYNOTE-564 trial demonstrated that patients with high-risk RCC treated with adjuvant pembrolizumab benefited from prolonged disease-free survival, although the extent of that benefit is unclear with maturing OS data [29]. As the role of adjuvant IO is evolving for early-stage RCC, there are unanswered questions as to how to treat these patients if disease were to recur. ICB-based combination strategies provide patients the greatest chance of response and greatest depth of response. However, the role of immunotherapy rechallenge in this setting is yet to be defined. We do not fully understand the inherent tumor resistance mechanisms for patients who develop recurrence while on adjuvant therapy (or shortly thereafter) and in those who have a prolonged disease-free interval prior to recurrence. As such, there is a new need for high-level prospective data in this arena. In the absence of such data, we will need to extrapolate from completed and ongoing trials in the post-ICI mRCC setting.

The phase III OMNIVORE trial was a carefully designed response-adapted study in which patients were treated with single-agent nivolumab followed by response-based ipilimumab as a salvage strategy. There was a remarkably low conversion rate of nivolumab non-responders, as only 2 of 57 patients treated with salvage IO converted to a partial response (while no CRs were observed) [73]. An additional relevant study is a phase Ib/II KEYNOTE-146 trial, which included a cohort assessing lenvatinib/pembrolizumab in ICB-pretreated patients. The study showed that salvage ICB-based combination therapy is feasible in this population, although with dampened efficacy compared to the treatment-naïve cohort [74]. There are ongoing studies (e.g., PDIGREE [NCT03793166], CONTACT-03 [NCT04338269], TiNivo-2 [NCT04987203]) which may provide further insight into optimal treatment approach here. Invariably, we anticipate and welcome carefully designed prospective studies for patients previously treated with adjuvant-ICB to better determine optimal treatment sequencing.

## 6. Ongoing Areas of Study within the Front-Line Treatment Paradigm

### 6.1. Predictive Biomarkers

There are no clinically validated predictive or prognostic biomarkers to guide treatment selection currently available. This remains an urgent and unmet need, as treatment responsiveness remains highly variable, and most patients will eventually develop resistance to treatment, let alone the risk of primary progression. Conventional biomarkers utilized in other immunogenic tumor types, such as PD-L1 and tumor mutational burden, do not show a consistent correlation with treatment outcomes across studies, albeit the studies were not uniformly designed and have significant limitations, hindering our interpretation of clinically utilizable data.

Single-gene biomarkers showed initial promise, with special attention to PBRM1, SMARCA4, and SETD2 among others. PBRM1 loss or mutation is prevalent in clear cell RCC and is associated with a nonimmunogenic tumor phenotype and possibly increased responsiveness to anti-angiogenesis therapy [75,76]. However, utility is limited as this association has yet to be established in large prospective randomized study. Single-gene biomarkers have not demonstrated replicable predictive ability in major studies. We now understand that transitioning from single-gene biomarkers to gene expression signatures may have greater impact. A correlative study from the IMmotion 151 trial established a transcriptomic map of enrolled patients. This study enrolled 915 IMDC all-risk patients to receive either atezolizumab/bevacizumab or sunitinib. The study did not meet its primary endpoint and failed to demonstrate an overall survival benefit to combination therapy, but its well-conceptualized exploratory analysis helped define the biology of mRCC. The analysis revealed seven molecular subtypes representing cluster patterns of underlying disease biology. Patient tumors characterized by immunogenic/inflammatory clusters (T-effector/proliferative, proliferative, and small nucleolar RNA transcription profiles) had improved survival outcomes when treated with ICB. However, patients with tumors enriched with angiogenesis clusters did not derive benefit from added IO to anti-angiogenesis [72].

The phase II BIONIKK study was the first trial to prospectively allocate patients to treatment groups based on a pre-defined 35-gene mRNA signature profile. Treatment arms included TKI monotherapy, ICB monotherapy, or dual-ICB combination therapy [77]. The study nicely demonstrated the feasibility of biomarker-based personalized treatment in mRCC and served as a prime example of the potential for biomarkers for ongoing trial development. Building upon BIONIKK, we are eagerly awaiting the phase II OPTIC trial, which is not yet recruiting (NCT05361720). OPTIC will employ the aforementioned biologic clusters and prospectively assign patients to IO/IO or IO/TKI combination therapy, depending on tumor genomics.

Despite this ongoing progress, we are still in need of further biomarker development with a focus on deciphering ideal TKI selection among the various IO/TKI regimens. There are certainly differences between axitinib, lenvatinib, and cabozantinib based on targets and further investigation into IO/TKI synergism and angiogenesis signatures may enable clinically impactful biomarker development in this space. Further, investigators are assessing alternative biomarkers for response to PD-1/PD-L1 therapy, although these remain grossly inconsistent and not relevant for clinical practice quite yet [78].

### 6.2. Immunomodulation

The RCC immune milieu is dynamic, and response to ICB is dependent on multiple factors that are not yet fully understood. The gut microbiome has a direct influence on the response to anti-PD-1 immunotherapy in cancer patients [79]. Interestingly, studies suggest an interplay between the microbiome and the RCC tumor microenvironment, with temporal changes in microbiota through ICB treatment course [80]. Taken together, there is the biologic rationale for further investigation of gut microbiome modulation to augment the immunogenicity of mRCC. In an open-label prospective study, patients were randomized in a 2:1 fashion to receive ipilimumab/nivolumab with or without daily oral CBM588 (a bifidogenic live bacterial product). Interestingly, the PFS was longer in patients receiving the oral bacterial product (12.7 months vs. 2.5 months, HR 0.15, 95% CI, 0.05–0.47, *p* = 0.001). ORR also favored the CBM588 treatment arm, although this result was not statistically significant (58% versus 20%, *p* = 0.06). There was no additional significant toxicity due to CBM588 [81]. These data warrant larger studies further exploring opportunities for immunomodulation in conjunction with both standard ICB and novel therapies.

### 6.3. Future Therapies and Ongoing Clinical Trials

Novel upstream targets within the VHL-HIF-VEGF axis are being investigated. Small-molecule inhibitors targeting HIF-2α are being developed, with a few under clinical investigation [82]. Belzutifan (MK-6482) is a second-generation HIF-2α inhibitor with remarkable efficacy in von Hippel–Lindau disease-associated RCC [83]. Naturally, MK-6482 is now under active investigation in sporadic mRCC. Favorable results from a phase I study in a heavily pretreated population have further increased enthusiasm toward this agent. Of 55 patients with clear cell mRCC across all IMDC risk groups, the ORR was 25%, and the DCR was 80%. Median PFS was 14.5 months (95% CI, 7.3–NR). These remarkable antitumor responses were in the setting of a manageable toxicity profile [84]. In the front-line setting, the open-label phase II MK-6482-003 study is actively recruiting patients to undergo treatment with a combination of belzutifan and cabozantinib. The primary endpoint is ORR, and secondary endpoints include PFS and OS. It will be interesting to see if combination therapy targeting multiple drivers within the VHL canonical pathway will have synergistic effects (NCT03634540) (Table 4).

Treatment intensification beyond doublet therapies may provide higher ORRs and deeper responses than currently approved options. The highly anticipated COSMIC-313 trial is a large randomized control, double-blind phase III study investigating triplet therapy with nivolumab/ipilimumab with or without cabozantinib in previously untreated IMDC intermediate-and poor-risk patients. Topline results reported that triplet therapy significantly reduced the risk of disease progression (HR for PFS, 0.73; 95% CI, 0.57–0.94; *p* = 0.01). Overall survival and full safety profiles are pending maturity, but it is possible that this trial will gain regulatory approval in the near future if mature results remain positive. Triplet therapy is also being investigated with belzutifan. The phase III MK-6848-012 trial is an elegantly designed randomized trial that plans to recruit 1,431 patients to receive lenvatinib/pembrolizumab, lenvatinib/pembrolizumab + belzutifan, or lenvatinib/pembrolizumab + quavonlimab, which is a novel CTLA-4 antibody (NCT04586231). Conversely, there are also prospective trials investigating treatment deintensification. The phase III CA209-8Y8 trial is investigating outcomes in previously untreated IMDC intermediate- or poor-risk patients when treated with ipilimumab/nivolumab combination therapy or nivolumab monotherapy (NCT03873402). The TIDE-A study is an elegantly designed phase II in which IO/TKI-treated patients discontinue their TKI and continue with IO maintenance (NCT04698213). This study follows the rationale that TKIs are particularly important for initial disease control, whereas IO has more long-term antitumor activity. Both these trials certainly have the potential to change clinical practice.

Novel IO-based strategies are under investigation, either to augment ICB or to potentiate alternative immune mechanisms. Bempegaldesleukin is a PEGylated IL-2 that recently gained FDA breakthrough designation in metastatic melanoma. It has been employed in the mRCC space as well, with early-phase I/II data demonstrating safety and efficacy in conjunction with nivolumab as first-line therapy in mRCC [85]. The subsequent larger phase III PIVOT-09 trial failed to meet the prespecified boundary for significance for ORR or OS, and the trial was discontinued. Final results are yet to be reported (NCT03729245).

As we further elucidate the molecular underpinnings of mRCC, we expect to see novel therapeutic targets emerge in prospective clinical studies. Epigenetic regulation is a fitting approach as histone deacetylases (HDACs) are overexpressed in RCC, with lower protein acetylation in non-cancerous renal tissue [86,87]. HDAC inhibitors abexinostat and vorinostat have both demonstrated antitumor activity with acceptable safety profiles when paired with anti-angiogenesis therapy in early-phase trials [88,89]. A randomized, phase III, double-blind, placebo-controlled study of pazopanib with or without abexinostat in previously untreated patients is now underway (NCT03592472).

Investigators continue to explore new approaches to RCC tumorigenesis in pre-clinical and early-phase studies. These include and are not limited to: metabolic reprogramming with a focus on RCC cancer cell metabolites, repurposing drugs already employed in other tumor settings such as CKD4/6 inhibitors and IDO1 inhibitors, and cellular therapy approaches. While we have enthusiasm for the ongoing study of these agents, it is too early to consider them as having practice-changing potential.

## 7. Conclusions

The management of clear cell RCC has undergone radical changes as we are furthering our understanding of the mRCC disease process and its underlying biologic drivers. We are witnessing a paradigm change in the approach to newly diagnosed metastatic disease. Careful attention to tumor biology and clinicopathologic patient characteristics can inform patient-centered decisions to either enter active surveillance or choose metastasis-directed therapy without the immediate need for upfront systemic therapies and their associated toxicities. Front-line treatment now typically employs IO/IO or various IO/TKI combination therapies. Although the landmark studies leading to each approval are similarly designed, there are distinct features to each therapy that may allow for a personalized approach to each patient accounting for comorbidities, patient values, and tumor characteristics. Ongoing studies identifying predictive biomarkers will provide clarity and provide objective measures to guide treatment choice in this crowded treatment landscape and usher us into an era of personalized medicine in mRCC. Integration of validated biomarkers into prospective studies evaluating novel therapies will be essential to achieving this goal.

## Figures and Tables

**Table 1:** Front-line approaches to metastatic RCC avoiding immediate initiation of systemic therapy.

Active Surveillance				
Study	Study Setting (sample size)	Patient population and characteristics	Median follow-up	Derived benefit (months)
Rini et al. [6]	Prospective phase II trial (*n* = 52)	Treatment-naive, asymptomatic, metastatic RCC. - 75% IMDC intermediate risk - 98% prior nephrectomy - 83% ≤ 2 sites of metastases	38.1 months	Median time on AS 14.9 m (95% CI, 10.6–25.0) Median OS from start of surveillance 44.5 m (95% CI, 37.6 m-NR)
MaRCC trial [7]	Prospective observational study (*n* = 504, 143 on AS)	Treatment-naïve metastatic RCC - 38% IMDC intermediate risk - 58% prior nephrectomy - 92% ≤ 2 sites of metastases	33.0 months	Median OS was NR (95% CI, 122 months—NE) Kaplan–Meier estimate for living at 3 years: 84%
Cytoreductive Nephrectomy				
Study	Study setting (sample size)	Trial design	Median Follow-up	Derived benefit (months)
CARMENA [8]	Randomized phase III, non-inferiority trial of clear cell RCC (*n* = 576) MSKCC poor risk 43%	Nephrectomy followed by sunitinib vs. Receive sunitinib alone	50.9 months	Median OS (95% CI) Upfront CN: 15.6 m (95% CI, 12.5–18.6) Median OS sunitinib alone: 19.8 m (95% CI, 15.6–24.8) HR 0.97 (0.79–1.19)
SURTIME [9]	Randomized phase III, superiority trial of clear cell RCC (*n* = 458) MSKCC poor risk 11%	Immediate CN followed by sunitinib vs. Treatment with 3 cycles of sunitinib followed by CN	3.3 years	Median OS upfront CN: 15.0 m (95% CI, 9.3–29.5) Median OS upfront sunitinib: 32.4 m (95% CI, 14.5–65.3) HR 0.57 (0.34–0.95)
Metastasis-Directed Therapy				
Study	Study setting (sample size)	Study design	Median Follow-up	Derived benefit (months)
SBRT [10]	Single-arm Phase II trial of metastatic clear cell RCC with ≤5 sites of metastases and prior nephrectomy (*n* = 30)	Treatment with SBRT (defined as ≤5 fractions with ≥7 Gy per fraction) to all lesions and maintained off systemic therapy	17.5 months	Median PFS survival was 22.7 months (95% CI, 10.4–NR) 1-year PFS 64% (95% CI, 48–85)
Metastasectomy [11]	Observational study of registry patients who underwent partial or radical nephrectomy with occurrence of metastasis treated with complete metastasectomy (*n* = 403, 147 with complete metastasectomy)	Associations of complete metastasectomy with cancer specific and OS were assessed in the era of TKI and ICB	3.9 years	Two-year cancer-specific survival was significantly greater in patients with vs. without complete metastasectomy (84% vs. 54%, *p* < 0.001) HR for death from RCC 0.47 (95% CI, 0.34–0.65, *p* < 0.001)

IMDC, International Metastatic RCC Database Consortium; RCC, renal cell carcinoma; AS, active surveillance; OS, overall survival; NR, not reached; NE, not evaluable; CN, cytoreductive nephrectomy; MSKCC, Memorial Sloan Kettering Cancer Center; SBRT, stereotactic body radiotherapy; PFS, progression-free survival; TKI, tyrosine kinase inhibitor; ICB, immune checkpoint blockade.

**Table 2:** Front-line immuno-oncology (IO)-based combination therapies in metastatic clear cell RCC with extended follow up-data.

	Intention-to-Treat Population						IMDC Risk Group					
							Favorable			Intermediate/Poor		
	mOS [HR (95% CI)]	mPFS [HR (95% CI)]	ORR (%)	Median DOR (Months)	CR (%)	Median TTR (Months)	mOS [HR (95% CI)]	mPFS [HR (95% CI)]	ORR (%)	mOS [HR (95% CI)]	mPFS [HR (95% CI)]	ORR (%)
Checkmate 214 ^#^ Ipi/Nivo vs. Sunitinib	56.0 vs. 38.0 [0.72 (0.62–0.85)]	12.0 vs. 12.0 [0.86 (0.73–1.01)]	39.12 vs. 32.3	NR vs. 25.0	10.7 vs. 2.6 *	2.8 vs. 4.0 *	74.0 vs. 68.0 [0.94 (0.65–1.37)]	12.4 vs. 28.9 [1.60 (1.1–2.3)]	30.1 vs. 52.6	47.0 vs. 27.0 [0.68 (0.58–0.81)]	12.0 vs. 8.0 [0.73 (0.61–0.87)]	42.1 vs. 27.2
KEYNOTE 426 ^##^ Pembro/Axi vs. Sunitinib	45.7 vs. 40.1 [0.73 (0.60–0.88)]	15.7 vs. 11.1 [0.68 (0.58–0.80)]	60.4 vs. 39.6	23.6 vs. 15.3	10.0 vs. 3.5	2.8 vs. 3.0	NR [1.17 (0.76–1.80)]	20.7 vs. 17.8 [0.76 (0.56–1.03)]	68.8 vs. 50.4	Not reported [0.64 (0.52–0.80)]	13.8 vs. 8.2 [0.67 (0.55–0.81)]	56.5 vs. 34.9
CheckMate 9ER Nivo/Cabo vs. Sunitinib	NR vs. 29.5 [0.66 (0.50–0.87)]	17.0 vs. 8.3 [0.52 (0.43–0.64)]	54.8 vs. 28.4	20.2 vs. 11.5	9.3 vs. 4.3	2.8 vs. 4.5	NR vs. NR [0.94 (0.46–1.92)]	24.7 vs. 12.8 [0.58 (0.36–0.93)]	66.2 vs. 44.4	Int NR vs. NR [0.74 (0.50–1.08)]	Int 17.5 vs. 8.5 [0.58 (0.45–0.76)]	Int 55.9 vs. 28.7
										Poor NR vs. 11.2 [0.45 (0.27–0.76)]	Poor 9.9 vs. 4.2 [0.36 (0.23–0.56)]	Poor 37.7 vs. 10.3
CLEAR Pembro/Lenvatinib vs. Sunitinib	NR vs. NR [0.66 (0.49–0.88)]	23.9 vs. 9.2 [0.39 (0.32–0.49)]	71.0 vs. 36.1	25.8 vs. 14.6	16.1 vs. 4.2	1.94 vs. 1.94	NR vs. NR [1.15 (0.55–2.40)]	28.1 vs. 12.9 [0.41 (0.28–0.62)]	68.2 vs. 50.8	NR vs. NR [0.58 (0.42–0.80)]	22.1 vs. 5.9 [0.36 (0.28–0.47)]	72.4 vs. 28.8

DOR, duration of response; TTR, Time to response; Ipi, Ipilimumab; Nivo, Nivolumab; Pembro, Pembrolizumab; Axi, Axitinib; Cabo, Cabozantinib; ORR, objective response rate; mPFS, median progression-free survival; HR, hazard ratio; NR, not reached; mOS, median overall survival. ^#^ Extended 5-year follow-up data, * Extended 4-year follow-up date, ^##^ Extended 3.5-year follow-up date.

**Table 3:** Summary of efficacy of ICB-based systemic therapy in mRCC with sarcomatoid features.

Trial	Sample Size	ORR (%)	CR (%)	PFS (Months)	HR PFS (95% CI)	OS (Months)	HR OS (95% CI)
CheckMate 214	Ipi + Nivo (*n* = 74)	61.0	19.0	26.5	0.54 (0.3–0.9)	NR	0.45 (0.3–0.7)
	Sunitinib (*n* = 65)	23.0	3.0	5.1		14.2	
KEYNOTE-426	Pembro + Axi (*n* = 46)	58.8	13.0	NR	0.52 (0.29–1.00)	NR	0.58 (0.21–1.59)
	Sunitinib (*n* = 50)	31.5	2.0	8.4		NR	
CheckMate 9ER	Nivo + Cabo (*n* = 34)	54.8	9.3	10.3	0.42 (0.23–0.74)	NR	0.36 (0.17–0.79)
	Sunitinib (*n* = 41)	28.4	4.3	4.2		19.7	
CLEAR	Pembro + Lenvatinib (*n* = 28)	60.7	NA	11.1	0.39 (0.18–0.84)	NR	0.91 (0.32–2.58)
	Sunitinib (*n* = 21)	23.8	NA	5.5		NR	

Ipi, ipilimumab; Nivo, Nivolumab; Sun, Sunitinib; Pembro, Pembrolizumab; ORR, objective response rate; CR, complete response; PFS, progression-free survival; HR, hazard ratio; OS, overall survival; NR, not reached; NA, not available.

**Table 4:** Future therapies and ongoing clinical trials.

Trial	Disease Setting	Comparator Arm	Treatment	Study Phase	Estimated Completion	Primary Endpoint(s)
NCT03937210 (COSMIC-313)	Previously untreated advanced or mRCC	Cabozantinib-matched placebo + Nivolumab + Ipilimumab	Cabozantinib + Nivolumab + Ipilimumab	Phase III	March 2025	PFS
NCT03873402 (CA209-8Y8)	Advanced RCC	N/A	Nivolumab + Ipilimumab	Phase III	March 2025	PFS, ORR
NCT03592472 (RENAVIV)	Locally advanced unrespectable or mRCC	Pazopanib + placebo	Pazopanib + Abexinostat	Phase III	June 2022	PFS
NCT04987203	Advanced RCC	Tivozanib	Tivozanib + Nivolumab;	Phase III	Aug 2025	PFS
NCT02811861 (CLEAR)	Advanced RCC	Sunitinib	*Arm A:* Lenvatinib + Everolimus *Arm B:* Lenvatinib + Pembrolizumab	Phase III	Oct 2024	PFS
NCT03729245	Untreated advanced RCC	Sunitinib or Cabozantinib	Bempegaldesleukin+ Nivolumab	Phase III	June 2024	ORR, OS
NCT04810078 (CheckMate-67T)	Advanced clear cell RCC	Intravenous Nivolumab	Subcutaneous Nivolumab	Phase III	June 2026	Time-averaged serum conc., trough serum concentration at steady state
NCT05239728	Clear cell RCC	Placebo + Pembrolizumab	Belzutifan + Pembrolizumab	Phase III	Jan 2030	DFS
NCT03873402	Advanced RCC	Nivolumab + Ipilimumab	Nivolumab	Phase III	March 2025	PFS, ORR
NCT03288532 (RAMPART)	Resected primary RCC at high or intermediate relapse risk	N/A	*Arm A:* Active monitoring *Arm B:* Durvalumab *Arm C:* Durvalumab + Tremelimumab	Phase III	Dec 2034	DFS, OS
NCT04394975	Advanced RCC	Sunitinib	Toripalimab + Axitinib	Phase III	June 2023	PFS
NCT03095040 (CONCEPT)	mRCC	Everolimus	Vorolanib + Everolimus	Phase III	Dec 2021	PFS
NCT04698213 (TIDE-A)	Untreated mRCC	N/A	Avelumab + Intermittent Axitinib	Phase II	Oct 2024	ORR
NCT04976634 (MK-6848-012)	Solid tumors	Pembrolizumab + Lenvatinib	Arm 1: Pembrolizumab + Belzutifan + Lenvatinib	Phase II	August 2026	DLT, AE, ORR
NCT03634540 (MK-6482-003)	Advanced clear cell RCC	N/A	Belzutifan + Cabozantinib	Phase II	August 2025	ORR
NCT04846920	Advanced clear cell RCC	N/A	Belzutifan	Phase I	July 2025	AE, DLT

PFS, progression-free survival; ORR, objective response rate; DLT, dose-limiting toxicity; RCC, renal cell carcinoma; ccRCC, clear cell renal cell carcinoma; AE, adverse events; OS, overall survival; DFS, disease-free survival; mRCC, metastatic renal cell carcinoma.

## References

[1] Padala S.A., Barsouk A., Thandra K.C., Saginala K., Mohammed A., Vakiti A., Rawla P., Barsouk A. (2020). Epidemiology of Renal Cell Carcinoma. World J. Oncol..

[2] Geynisman D.M., Maranchie J.K., Ball M.W., Bratslavsky G., Singer E.A. (2021). A 25 year perspective on the evolution and advances in an understanding of the biology, evaluation and treatment of kidney cancer. Urol. Oncol..

[3] Keith B., Johnson R.S., Simon M.C. (2011). HIF1alpha and HIF2alpha: Sibling rivalry in hypoxic tumour growth and progression. Nat. Rev. Cancer.

[4] Nickerson M.L., Jaeger E., Shi Y., Durocher J.A., Mahurkar S., Zaridze D., Matveev V., Janout V., Kollarova H., Bencko V. (2008). Improved identification of von Hippel-Lindau gene alterations in clear cell renal tumors. Clin. Cancer Res..

[5] Tran J., Ornstein M.C. (2022). Clinical Review on the Management of Metastatic Renal Cell Carcinoma. JCO Oncol. Pract..

[6] Rini B.I., Dorff T.B., Elson P., Rodriguez C.S., Shepard D., Wood L., Humbert J., Pyle L., Wong Y.N., Finke J.H. (2016). Active surveillance in metastatic renal-cell carcinoma: A prospective, phase 2 trial. Lancet Oncol..

[7] Harrison M.R., Costello B.A., Bhavsar N.A., Vaishampayan U., Pal S.K., Zakharia Y., Jim H.S.L., Fishman M.N., Molina A.M., Kyriakopoulos C.E. (2021). Active surveillance of metastatic renal cell carcinoma: Results from a prospective observational study (MaRCC). Cancer.

[8] Mickisch G.H., Garin A., van Poppel H., de Prijck L., Sylvester R. (2001). Radical nephrectomy plus interferon-alfa-based immunotherapy compared with interferon alfa alone in metastatic renal-cell carcinoma: A randomised trial. Lancet.

[9] Mejean A., Ravaud A., Thezenas S., Colas S., Beauval J.B., Bensalah K., Geoffrois L., Thiery-Vuillemin A., Cormier L., Lang H. (2018). Sunitinib Alone or after Nephrectomy in Metastatic Renal-Cell Carcinoma. N. Engl. J. Med..

[10] Tang C., Msaouel P., Hara K., Choi H., Le V., Shah A.Y., Wang J., Jonasch E., Choi S., Nguyen Q.N. (2021). Definitive radiotherapy in lieu of systemic therapy for oligometastatic renal cell carcinoma: A single-arm, single-centre, feasibility, phase 2 trial. Lancet Oncol..

[11] Lyon T.D., Thompson R.H., Shah P.H., Lohse C.M., Boorjian S.A., Costello B.A., Cheville J.C., Leibovich B.C. (2020). Complete Surgical Metastasectomy of Renal Cell Carcinoma in the Post-Cytokine Era. J. Urol..

[12] Heng D.Y., Xie W., Regan M.M., Harshman L.C., Bjarnason G.A., Vaishampayan U.N., Mackenzie M., Wood L., Donskov F., Tan M.H. (2013). External validation and comparison with other models of the International Metastatic Renal-Cell Carcinoma Database Consortium prognostic model: A population-based study. Lancet Oncol..

[13] Lalani A.A., Heng D.Y.C., Basappa N.S., Wood L., Iqbal N., McLeod D., Soulieres D., Kollmannsberger C. (2022). Evolving landscape of first-line combination therapy in advanced renal cancer: A systematic review. Ther. Adv. Med. Oncol..

[14] Fitzgerald K.N., Lee C.H. (2022). Personalizing First-Line Management of Metastatic Renal Cell Carcinoma: Leveraging Current and Novel Therapeutic Options. J. Natl. Compr. Cancer Netw..

[15] Ernst M.S., Navani V., Wells J.C., Donskov F., Basappa N.S., Labaki C., Pal S.K., Meza L.A., Wood L., Ernst D.S. (2022). Characterizing IMDC prognostic groups in contemporary first-line combination therapies for metastatic renal cell carcinoma (mRCC). J. Clin. Oncol..

[16] Martini D.J., Liu Y., Shabto J.M., Carthon B.C., Hitron E.E., Russler G.A., Caulfield S., Kissick H.T., Harris W.B., Kucuk O. (2020). Novel Risk Scoring System for Patients with Metastatic Renal Cell Carcinoma Treated with Immune Checkpoint Inhibitors. Oncologist.

[17] Voss M.H., Reising A., Cheng Y., Patel P., Marker M., Kuo F., Chan T.A., Choueiri T.K., Hsieh J.J., Hakimi A.A. (2018). Genomically annotated risk model for advanced renal-cell carcinoma: A retrospective cohort study. Lancet Oncol..

[18] Flanigan R.C., Salmon S.E., Blumenstein B.A., Bearman S.I., Roy V., McGrath P.C., Caton J.R., Munshi N., Crawford E.D. (2001). Nephrectomy followed by interferon alfa-2b compared with interferon alfa-2b alone for metastatic renal-cell cancer. N. Engl. J. Med..

[19] Bex A., Mulders P., Jewett M., Wagstaff J., van Thienen J.V., Blank C.U., van Velthoven R., Del Pilar Laguna M., Wood L., van Melick H.H.E. (2019). Comparison of Immediate vs Deferred Cytoreductive Nephrectomy in Patients with Synchronous Metastatic Renal Cell Carcinoma Receiving Sunitinib: The SURTIME Randomized Clinical Trial. JAMA Oncol..

[20] Mejean A., Ravaud A., Thezenas S., Chevreau C., Bensalah K., Geoffrois L., Thiery-Vuillemin A., Cormier L., Lang H., Guy L. (2021). Sunitinib Alone or After Nephrectomy for Patients with Metastatic Renal Cell Carcinoma: Is There Still a Role for Cytoreductive Nephrectomy?. Eur. Urol..

[21] Bhindi B., Graham J., Wells J.C., Bakouny Z., Donskov F., Fraccon A., Pasini F., Lee J.L., Basappa N.S., Hansen A. (2020). Deferred Cytoreductive Nephrectomy in Patients with Newly Diagnosed Metastatic Renal Cell Carcinoma. Eur. Urol..

[22] de Bruijn R., Wimalasingham A., Szabados B., Stewart G.D., Welsh S.J., Kuusk T., Blank C., Haanen J., Klatte T., Staehler M. (2020). Deferred Cytoreductive Nephrectomy Following Presurgical Vascular Endothelial Growth Factor Receptor-targeted Therapy in Patients with Primary Metastatic Clear Cell Renal Cell Carcinoma: A Pooled Analysis of Prospective Trial Data. Eur. Urol. Oncol..

[23] Ghatalia P., Handorf E.A., Geynisman D.M., Deng M., Zibelman M.R., Abbosh P., Anari F., Greenberg R.E., Viterbo R., Chen D. (2022). The Role of Cytoreductive Nephrectomy in Metastatic Renal Cell Carcinoma: A Real-World Multi-Institutional Analysis. J. Urol..

[24] Ruhle A., Andratschke N., Siva S., Guckenberger M. (2019). Is there a role for stereotactic radiotherapy in the treatment of renal cell carcinoma?. Clin. Transl. Radiat. Oncol..

[25] De La Pinta C., Latorre R.G., Fuentes R. (2022). SBRT in Localized Renal Carcinoma: A Review of the Literature. Anticancer Res..

[26] Zaorsky N.G., Lehrer E.J., Kothari G., Louie A.V., Siva S. (2019). Stereotactic ablative radiation therapy for oligometastatic renal cell carcinoma (SABR ORCA): A meta-analysis of 28 studies. Eur. Urol. Oncol..

[27] Malleo G., Salvia R., Maggino L., Marchegiani G., D’Angelica M., DeMatteo R., Kingham P., Pulvirenti A., Sereni E., Jarnagin W.R. (2021). Long-term Outcomes After Surgical Resection of Pancreatic Metastases from Renal Clear-Cell Carcinoma. Ann. Surg. Oncol..

[28] Benamran D., Albiges L., Bex A., Giannarini G., Capitanio U., Roupret M. (2022). Treatment Options for De Novo Metastatic Clear-cell Renal Cell Carcinoma: Current Recommendations and Future Insights. Eur. Urol. Oncol..

[29] Choueiri T.K., Tomczak P., Park S.H., Venugopal B., Ferguson T., Chang Y.H., Hajek J., Symeonides S.N., Lee J.L., Sarwar N. (2021). Adjuvant Pembrolizumab after Nephrectomy in Renal-Cell Carcinoma. N. Engl. J. Med..

[30] Bristol Myers Squibb (2022). Bristol Myers Squibb Provides Update on CheckMate-914 Trial Evaluating Opdivo (nivolumab) Plus Yervoy (ipilimumab) as Adjuvant Treatment of Localized Renal Cell Carcinoma.

[31] Plieth J. Two Adjuvant Kidney Cancer Failures Embolden Keytruda. https://www.evaluate.com/vantage/articles/news/trial-results-snippets/two-adjuvant-kidney-cancer-failures-embolden-keytruda.

[32] Motzer R.J., Tannir N.M., McDermott D.F., Aren Frontera O., Melichar B., Choueiri T.K., Plimack E.R., Barthelemy P., Porta C., George S. (2018). Nivolumab plus Ipilimumab versus Sunitinib in Advanced Renal-Cell Carcinoma. N. Engl. J. Med..

[33] Motzer R., Tannir N., McDermott D., Burotto M., Choueiri T., Hammers H., Plimack E., Porta C., George S., Powles T. Conditional Survival and 5-Year Follow-Up in CheckMate 214: First-Line Nivolumab + Ipilimumab (N+I) Versus Sunitinib (S) in Advanced Renal Cell Carcinoma (aRCC). Proceedings of the European Society for Medical Oncology.

[34] Yi M., Jiao D., Qin S., Chu Q., Wu K., Li A. (2019). Synergistic effect of immune checkpoint blockade and anti-angiogenesis in cancer treatment. Mol. Cancer.

[35] Rini B.I., Plimack E.R., Stus V., Gafanov R., Hawkins R., Nosov D., Pouliot F., Alekseev B., Soulieres D., Melichar B. (2019). Pembrolizumab plus Axitinib versus Sunitinib for Advanced Renal-Cell Carcinoma. N. Engl. J. Med..

[36] Powles T., Plimack E.R., Soulieres D., Waddell T., Stus V., Gafanov R., Nosov D., Pouliot F., Melichar B., Vynnychenko I. (2020). Pembrolizumab plus axitinib versus sunitinib monotherapy as first-line treatment of advanced renal cell carcinoma (KEYNOTE-426): Extended follow-up from a randomised, open-label, phase 3 trial. Lancet Oncol..

[37] Albiges L., Tannir N.M., Burotto M., McDermott D., Plimack E.R., Barthelemy P., Porta C., Powles T., Donskov F., George S. (2020). Nivolumab plus ipilimumab versus sunitinib for first-line treatment of advanced renal cell carcinoma: Extended 4-year follow-up of the phase III CheckMate 214 trial. ESMO Open.

[38] Motzer R., Alekseev B., Rha S.Y., Porta C., Eto M., Powles T., Grunwald V., Hutson T.E., Kopyltsov E., Mendez-Vidal M.J. (2021). Lenvatinib plus Pembrolizumab or Everolimus for Advanced Renal Cell Carcinoma. N. Engl. J. Med..

[39] Motzer R.J., Penkov K., Haanen J., Rini B., Albiges L., Campbell M.T., Venugopal B., Kollmannsberger C., Negrier S., Uemura M. (2019). Avelumab plus Axitinib versus Sunitinib for Advanced Renal-Cell Carcinoma. N. Engl. J. Med..

[40] Haanen J.B.A.G., Larkin J., Choueiri T.K., Albiges L., Rini B.I., Atkins M.B., Schmidinger M., Penkov K., Thomaidou D., Wang J. (2021). Efficacy of avelumab + axitinib (A + Ax) versus sunitinib (S) by IMDC risk group in advanced renal cell carcinoma (aRCC): Extended follow-up results from JAVELIN Renal 101. J. Clin. Oncol..

[41] Zibelman M.R., Carducci M.A., Ged Y., Molina A.M., Ravilla R., Shaffer D.R., Lambert C., Tafseer M., Basiura R., Weismann D. (2022). A phase I/II study of nivolumab and axitinib in patients with advanced renal cell carcinoma. J. Clin. Oncol..

[42] Pichler R., Comperat E., Klatte T., Pichler M., Loidl W., Lusuardi L., Schmidinger M. (2019). Renal Cell Carcinoma with Sarcomatoid Features: Finally New Therapeutic Hope?. Cancers.

[43] Blum K.A., Gupta S., Tickoo S.K., Chan T.A., Russo P., Motzer R.J., Karam J.A., Hakimi A.A. (2020). Sarcomatoid renal cell carcinoma: Biology, natural history and management. Nat. Rev. Urol..

[44] Tannir N.M., Signoretti S., Choueiri T.K., McDermott D.F., Motzer R.J., Flaifel A., Pignon J.C., Ficial M., Frontera O.A., George S. (2021). Efficacy and Safety of Nivolumab Plus Ipilimumab versus Sunitinib in First-line Treatment of Patients with Advanced Sarcomatoid Renal Cell Carcinoma. Clin. Cancer Res..

[45] Iacovelli R., Ciccarese C., Bria E., Bracarda S., Porta C., Procopio G., Tortora G. (2020). Patients with sarcomatoid renal cell carcinoma—redefining the first-line of treatment: A meta-analysis of randomised clinical trials with immune checkpoint inhibitors. Eur. J. Cancer.

[46] Motzer R.J., Jonasch E., Boyle S., Carlo M.I., Manley B., Agarwal N., Alva A., Beckermann K., Choueiri T.K., Costello B.A. (2020). NCCN Guidelines Insights: Kidney Cancer, Version 1.2021. J. Natl. Compr. Cancer Netw..

[47] Cella D., Grunwald V., Escudier B., Hammers H.J., George S., Nathan P., Grimm M.O., Rini B.I., Doan J., Ivanescu C. (2019). Patient-reported outcomes of patients with advanced renal cell carcinoma treated with nivolumab plus ipilimumab versus sunitinib (CheckMate 214): A randomised, phase 3 trial. Lancet Oncol..

[48] Wilson L.E., Spees L., Pritchard J., Greiner M.A., Scales C.D., Baggett C.D., Kaye D., George D.J., Zhang T., Wheeler S.B. (2021). Real-World Utilization of Oral Anticancer Agents and Related Costs in Older Adults with Metastatic Renal Cell Carcinoma in the United States. Kidney Cancer.

[49] Chan A., Dang C., Wisniewski J., Weng X., Hynson E., Zhong L., Wilson L. (2022). A Cost-effectiveness Analysis Comparing Pembrolizumab-Axitinib, Nivolumab-Ipilimumab, and Sunitinib for Treatment of Advanced Renal Cell Carcinoma. Am. J. Clin. Oncol..

[50] Geynisman D.M., Du E.X., Yang X., Sendhil S.R., Tejo V.D., Betts K.A., Huo S. (2022). Temporal trends of adverse events and costs of nivolumab plus ipilimumab versus sunitinib in advanced renal cell carcinoma. Future Oncol..

[51] Rathmell W.K., Rumble R.B., Van Veldhuizen P.J., Al-Ahmadie H., Emamekhoo H., Hauke R.J., Louie A.V., Milowsky M.I., Molina A.M., Rose T.L. (2022). Management of Metastatic Clear Cell Renal Cell Carcinoma: ASCO Guideline. J. Clin. Oncol..

[52] Fan Z., Huang Z., Huang X. (2021). Bone Metastasis in Renal Cell Carcinoma Patients: Risk and Prognostic Factors and Nomograms. J. Oncol..

[53] Escudier B., Powles T., Motzer R.J., Olencki T., Aren Frontera O., Oudard S., Rolland F., Tomczak P., Castellano D., Appleman L.J. (2018). Cabozantinib, a New Standard of Care for Patients with Advanced Renal Cell Carcinoma and Bone Metastases? Subgroup Analysis of the METEOR Trial. J. Clin. Oncol..

[54] Apolo A.B., Powles T., Burotto M., Bourlon M.T., Hsieh J.J., Basso U., Shah A.Y., Suarez C., Porta C., Barrios C.H. Nivolumab Plus Cabozantinib vs. Sunitinib in Patients with Advanced Renal Cell Carcinoma and Bone Metastasis: Subgroup Analysis of the Phase 3 CheckMate 9ER Trial. Proceedings of the 2021 International Kidney Cancer Symposium.

[55] Zhang Y., Guessous F., Kofman A., Schiff D., Abounader R. (2010). XL-184, a MET, VEGFR-2 and RET kinase inhibitor for the treatment of thyroid cancer, glioblastoma multiforme and NSCLC. IDrugs.

[56] Hirsch L., Martinez Chanza N., Farah S., Xie W., Flippot R., Braun D.A., Rathi N., Thouvenin J., Collier K.A., Seront E. (2021). Clinical Activity and Safety of Cabozantinib for Brain Metastases in Patients with Renal Cell Carcinoma. JAMA Oncol..

[57] Choueiri T.K., Powles T., Burotto M., Escudier B., Bourlon M.T., Zurawski B., Oyervides Juarez V.M., Hsieh J.J., Basso U., Shah A.Y. (2021). Nivolumab plus Cabozantinib versus Sunitinib for Advanced Renal-Cell Carcinoma. N. Engl. J. Med..

[58] Schmidinger M., Danesi R., Jones R., McDermott R., Pyle L., Rini B., Negrier S. (2018). Individualized dosing with axitinib: Rationale and practical guidance. Future Oncol..

[59] Chen Y.W., Rini B.I. (2022). Approaches to First-Line Therapy for Metastatic Clear Cell Renal Cell Carcinoma. Curr. Oncol. Rep..

[60] Powles T. (2020). Treatment Choices for Front-line Metastatic Clear Cell Renal Cancer. Eur. Urol..

[61] Sullivan K.M., Keyes-Elstein L. (2020). Cross-trial comparisons in reviews: Proceed with caution. Nat. Rev. Rheumatol..

[62] Zhang X., Fu S., Meng R., Ren Y., Shang Y., Tian L. (2020). Is there an efficacy-effectiveness gap between randomized controlled trials and real-world studies in colorectal cancer: A systematic review and meta-analysis. Transl. Cancer Res..

[63] Singal A.G., Higgins P.D., Waljee A.K. (2014). A primer on effectiveness and efficacy trials. Clin. Transl. Gastroenterol..

[64] Zarrabi K.H.E., Miron B., Zibelman M., Anari F., Ghatalia P., Plimack E., Geynisman D. Comparative Effectiveness of Front-line Ipilimumab and Nivolumab or Axitinib and Pembrolizumab in Metastatic Clear Cell Renal Cell Carcinoma. Oncology.

[65] Gan C.L., Dudani S., Wells J.C., Schmidt A.L., Bakouny Z., Szabados B., Parnis F., Wong S., Lee J.-L., de Velasco G. (2021). Outcomes of first-line (1L) immuno-oncology (IO) combination therapies in metastatic renal cell carcinoma (mRCC): Results from the International mRCC Database Consortium (IMDC). J. Clin. Oncol..

[66] Phillips C.M., Parmar A., Guo H., Schwartz D., Isaranuwatchai W., Beca J., Dai W., Arias J., Gavura S., Chan K.K.W. (2020). Assessing the efficacy-effectiveness gap for cancer therapies: A comparison of overall survival and toxicity between clinical trial and population-based, real-world data for contemporary parenteral cancer therapeutics. Cancer.

[67] Regan M.M., Werner L., Rao S., Gupte-Singh K., Hodi F.S., Kirkwood J.M., Kluger H.M., Larkin J., Postow M.A., Ritchings C. (2019). Treatment-Free Survival: A Novel Outcome Measure of the Effects of Immune Checkpoint Inhibition-A Pooled Analysis of Patients with Advanced Melanoma. J. Clin. Oncol..

[68] Regan M.M., Jegede O.A., Mantia C.M., Powles T., Werner L., Motzer R.J., Tannir N.M., Lee C.H., Tomita Y., Voss M.H. (2021). Treatment-free Survival after Immune Checkpoint Inhibitor Therapy versus Targeted Therapy for Advanced Renal Cell Carcinoma: 42-Month Results of the CheckMate 214 Trial. Clin. Cancer Res..

[69] Motzer R.J., Tannir N.M., McDermott D.F. Conditional Survival and 5-Year Follow-Up in CheckMate 214: First-Line Nivolumab Plus Ipilimumab versus Sunitinib in Advanced Renal Cell Carcinoma. Proceedings of the International Kidney Cancer Symposium (IKCS).

[70] Atkins M.B., Jegede O.A., Haas N.B., McDermott D.F., Bilen M.A., Stein M., Sosman J.A., Alter R., Plimack E.R., Ornstein M. (2022). Phase II Study of Nivolumab and Salvage Nivolumab/Ipilimumab in Treatment-Naive Patients with Advanced Clear Cell Renal Cell Carcinoma (HCRN GU16-260-Cohort A). J. Clin. Oncol..

[71] Tykodi S.S., Donskov F., Lee J.-L., Szczylik C., Malik J., Alekseev B.Y., Larkin J.M.G., Matveev V.B., Gafanov R., Tomczak P. (2019). First-line pembrolizumab (pembro) monotherapy in advanced clear cell renal cell carcinoma (ccRCC): Updated results for KEYNOTE-427 cohort A. J. Clin. Oncol..

[72] Motzer R.J., Powles T., Atkins M.B., Escudier B., McDermott D.F., Alekseev B.Y., Lee J.L., Suarez C., Stroyakovskiy D., De Giorgi U. (2022). Final Overall Survival and Molecular Analysis in IMmotion151, a Phase 3 Trial Comparing Atezolizumab Plus Bevacizumab vs Sunitinib in Patients with Previously Untreated Metastatic Renal Cell Carcinoma. JAMA Oncol..

[73] McKay R.R., McGregor B.A., Xie W., Braun D.A., Wei X., Kyriakopoulos C.E., Zakharia Y., Maughan B.L., Rose T.L., Stadler W.M. (2020). Optimized Management of Nivolumab and Ipilimumab in Advanced Renal Cell Carcinoma: A Response-Based Phase II Study (OMNIVORE). J. Clin. Oncol..

[74] Lee C.H., Shah A.Y., Rasco D., Rao A., Taylor M.H., Di Simone C., Hsieh J.J., Pinto A., Shaffer D.R., Girones Sarrio R. (2021). Lenvatinib plus pembrolizumab in patients with either treatment-naive or previously treated metastatic renal cell carcinoma (Study 111/KEYNOTE-146): A phase 1b/2 study. Lancet Oncol..

[75] Braun D.A., Ishii Y., Walsh A.M., Van Allen E.M., Wu C.J., Shukla S.A., Choueiri T.K. (2019). Clinical Validation of PBRM1 Alterations as a Marker of Immune Checkpoint Inhibitor Response in Renal Cell Carcinoma. JAMA Oncol..

[76] Liu X.D., Kong W., Peterson C.B., McGrail D.J., Hoang A., Zhang X., Lam T., Pilie P.G., Zhu H., Beckermann K.E. (2020). PBRM1 loss defines a nonimmunogenic tumor phenotype associated with checkpoint inhibitor resistance in renal carcinoma. Nat. Commun..

[77] Vano Y.A., Elaidi R., Bennamoun M., Chevreau C., Borchiellini D., Pannier D., Maillet D., Gross-Goupil M., Tournigand C., Laguerre B. (2022). Nivolumab, nivolumab-ipilimumab, and VEGFR-tyrosine kinase inhibitors as first-line treatment for metastatic clear-cell renal cell carcinoma (BIONIKK): A biomarker-driven, open-label, non-comparative, randomised, phase 2 trial. Lancet Oncol..

[78] Sarkis J., Assaf J., Alkassis M. (2021). Biomarkers in renal cell carcinoma: Towards a more selective immune checkpoint inhibition. Transl. Oncol..

[79] Routy B., Le Chatelier E., Derosa L., Duong C.P.M., Alou M.T., Daillere R., Fluckiger A., Messaoudene M., Rauber C., Roberti M.P. (2018). Gut microbiome influences efficacy of PD-1-based immunotherapy against epithelial tumors. Science.

[80] Salgia N.J., Bergerot P.G., Maia M.C., Dizman N., Hsu J., Gillece J.D., Folkerts M., Reining L., Trent J., Highlander S.K. (2020). Stool Microbiome Profiling of Patients with Metastatic Renal Cell Carcinoma Receiving Anti-PD-1 Immune Checkpoint Inhibitors. Eur. Urol..

[81] Dizman N., Meza L., Bergerot P., Alcantara M., Dorff T., Lyou Y., Frankel P., Cui Y., Mira V., Llamas M. (2022). Nivolumab plus ipilimumab with or without live bacterial supplementation in metastatic renal cell carcinoma: A randomized phase 1 trial. Nat. Med..

[82] Choi W.S.W., Boland J., Lin J. (2021). Hypoxia-Inducible Factor-2alpha as a Novel Target in Renal Cell Carcinoma. J. Kidney Cancer VHL.

[83] Jonasch E., Donskov F., Iliopoulos O., Rathmell W.K., Narayan V.K., Maughan B.L., Oudard S., Else T., Maranchie J.K., Welsh S.J. (2021). Belzutifan for Renal Cell Carcinoma in von Hippel-Lindau Disease. N. Engl. J. Med..

[84] Choueiri T.K., Bauer T.M., Papadopoulos K.P., Plimack E.R., Merchan J.R., McDermott D.F., Michaelson M.D., Appleman L.J., Thamake S., Perini R.F. (2021). Inhibition of hypoxia-inducible factor-2alpha in renal cell carcinoma with belzutifan: A phase 1 trial and biomarker analysis. Nat. Med..

[85] Tannir N.M., Cho D.C., Diab A., Sznol M., Bilen M.A., Balar A.V., Grignani G., Puente E., Tang L., Chien D. (2022). Bempegaldesleukin plus nivolumab in first-line renal cell carcinoma: Results from the PIVOT-02 study. J. Immunother. Cancer.

[86] Kim I.H., Lee H.J. (2022). The Frontline Immunotherapy-Based Treatment of Advanced Clear Cell Renal Cell Carcinoma: Current Evidence and Clinical Perspective. Biomedicines.

[87] Kiweler N., Brill B., Wirth M., Breuksch I., Laguna T., Dietrich C., Strand S., Schneider G., Groner B., Butter F. (2018). The histone deacetylases HDAC1 and HDAC2 are required for the growth and survival of renal carcinoma cells. Arch. Toxicol..

[88] Aggarwal R., Thomas S., Pawlowska N., Bartelink I., Grabowsky J., Jahan T., Cripps A., Harb A., Leng J., Reinert A. (2017). Inhibiting Histone Deacetylase as a Means to Reverse Resistance to Angiogenesis Inhibitors: Phase I Study of Abexinostat Plus Pazopanib in Advanced Solid Tumor Malignancies. J. Clin. Oncol..

[89] Pili R., Liu G., Chintala S., Verheul H., Rehman S., Attwood K., Lodge M.A., Wahl R., Martin J.I., Miles K.M. (2017). Combination of the histone deacetylase inhibitor vorinostat with bevacizumab in patients with clear-cell renal cell carcinoma: A multicentre, single-arm phase I/II clinical trial. Br. J. Cancer.

